# Gait Segmentation Method Using a Plantar Pressure Measurement System with Custom-Made Capacitive Sensors

**DOI:** 10.3390/s20030656

**Published:** 2020-01-24

**Authors:** Pablo Aqueveque, Enrique Germany, Rodrigo Osorio, Francisco Pastene

**Affiliations:** Department of Electrical Engineering, Faculty of Engineering, Universidad de Concepción, 219 Edmundo Larenas St., 4030000 Concepción, Chile; enrique.germany@biomedica.udec.cl (E.G.); rodrigo.osorio@biomedica.udec.cl (R.O.); francisco.pastene@biomedica.udec.cl (F.P.)

**Keywords:** capacitive sensors, gait analysis, gait segmentation, pressure sensors

## Abstract

Gait analysis has been widely studied by researchers due to the impact in clinical fields. It provides relevant information on the condition of a patient’s pathologies. In the last decades, different gait measurement methods have been developed in order to identify parameters that can contribute to gait cycles. Analyzing those parameters, it is possible to segment and identify different phases of gait cycles, making these studies easier and more accurate. This paper proposes a simple gait segmentation method based on plantar pressure measurement. Current methods used by researchers and clinicians are based on multiple sensing devices (e.g., multiple cameras, multiple inertial measurement units (IMUs)). Our proposal uses plantar pressure information from only two sensorized insoles that were designed and implemented with eight custom-made flexible capacitive sensors. An algorithm was implemented to calculate gait parameters and segment gait cycle phases and subphases. Functional tests were performed in six healthy volunteers in a 10 m walking test. The designed in-shoe insole presented an average power consumption of 44 mA under operation. The system segmented the gait phases and sub-phases in all subjects. The calculated percentile distribution between stance phase time and swing phase time was almost 60%/40%, which is aligned with literature reports on healthy subjects. Our results show that the system achieves a successful segmentation of gait phases and subphases, is capable of reporting COP velocity, double support time, cadence, stance phase time percentage, swing phase time percentage, and double support time percentage. The proposed system allows for the simplification of the assessment method in the recovery process for both patients and clinicians.

## 1. Introduction

The inability to walk normally or walking with erratic patterns in gait manifests direct impacts on life quality, making a person less independent or even generating other health issues. An erratic gait is characterized by asymmetrical patterns that may cause pain or injuries to the back, hips, or lower limbs [1]. For the reasons mentioned above, gait analysis is of high relevance in clinical studies. Human gait analyses investigate human locomotion with the objective of determining correct functionality and abnormalities if there are any. The objective is to analyze gait patterns and classify them in order to guide the best diagnosis and treatment for patients [2]. In the last years, human gait analysis tools have generated huge changes in the treatment and evaluation processes of certain illnesses, as they enable faster and more accurate analyses of the human gait, detecting abnormal patterns and giving accurate follow-up indexes during treatment [3]. In the last decades, technological gait analysis tools, such as camera-based devices, inertial measurement devices, and pressure-sensing devices, have gained increasing importance, being massively adopted in different health center facilities. These tools enable high-precision analyses and aid in the diagnosis of diseases such as Parkinson’s, cerebral palsy, multiple sclerosis, and stroke [4].

In cases were technological tools are absent, clinicians performs gait analysis by bare observation of patients in standardized motor tests. Even though this is a good source of information, it relies on the clinician’s experience and lacks numerical objectivity when comparing between different clinicians, since the majority of motor skill tests are based on qualitative indexes. Modern equipment aids human gait analyses by allowing for the performance of gait cycles in specialized gait laboratories. These laboratories have expensive video equipment and optical markers for body segment tracking. These analyses are much more consistent as they provide objective numerical information, such as plantar pressure, weight distribution, gait phases, timing, and step length, among others. The disadvantages of these methods include the reliance on expensive equipment, periodic maintenance, the use of sometimes uncomfortable equipment by the patient, and the loss of generalization and the representation of real environment situations since measurement conditions in the laboratory are not identical to real-life situations and terrains [1,2,3].

For the reasons mentioned above, there is a huge interest in the design of low-cost, wearable devices to measure and track human movement in a comfortable way for the patient and with the capability of measurement in unrestricted environments. These wearable devices are achieving fast and easy human movement analysis based on objective and repeatable measurements. Wearable devices may also benefit cases were disease requires continuous monitoring during daily-life conditions for longer periods of time, which is not achievable though video analysis tools [2]. In the last decades, interest in wearable devices had increased significantly, and has led to the development of very simple devices capable of logging objective data indexes from human gait analyses. The denominated in-shoe devices are wearable devices capable of measuring plantar pressure from within the footwear, making it possible to obtain precise measurements even when using shoes. Some applications and development of capacitive sensors for plantar pressure sensing are described in the literature. One of the first approaches to capacitive sensor design for this type of application was described in [5]. These systems make it possible to measure dynamic pressures while the wearer walks or runs, logging data, making real-time analyses, and communicating information using wireless communication protocols such as Bluetooth or WiFi standards. Until now, available devices with these characteristics have been very expensive (with prices over USD 10,000). In addition, commercially available devices are not sufficiently miniaturized, having the electronics somewhere in the shoe, which affects normal gait patterns and compromises the data quality [1,2,4]. Some research papers describe textile implementations for actigraphy and gait analysis. These works show the development and implementation of smart socks with embedded pressure and strain sensors [6,7,8,9,10,11,12], and they have shown results in movement classification. However, embedding electronics in a sock usually take place outside the textile structure, which makes it uncomfortable to the user and could affect normal gait conditions. In addition, as the sensors are in an unstable textile structure, they are more susceptible to noise, requiring more powerful processing to filter and extract usable information from signals. Reviewed capacitive measurement systems do not address embedding capabilities, resulting in bulky devices which in the majority of cases have a direct impact on gait patterns.

In this paper, we propose a new in-shoe embedded system composed of an instrumented shoe insole with custom-made capacitive pressure sensors capable of performing automated human gait segmentation and analyses. The sensors are placed over the most anatomically representative spots in the foot. The proposed system embeds all electronic components within the insole, making the system more comfortable and easy to use.

## 2. Human Gait Analysis

The human gait is defined as a locomotion method generated by the use of both legs in an alternated way, allowing the support and propulsion of the human body. During walking, the gait can be divided into phases [13]. Gait execution requires the presence of periodic movements of each foot toward a support position and reaction forces applied to the feet, enough to give support to the body even under distortions due to pathological conditions [14]. The human gait can be studied due to its cyclical nature, which is known in the literature as the “gait cycle”. This condition allows the identification of normal and pathological patterns [13,14]. The gait cycle can be defined as the interval between two successive repetitive actions during the gait [13]. Defining a start- and end-point in the gait cycle allows us to generate intermediate divisions. There are eight events recognized during the execution of the human gait [14,15]:

1. Heel strike: considered the starting and finishing event of a gait cycle. Corresponds to the first contact between the heel and the ground.
2. Foot flat: The plantar surface touches the ground.
3. Midstance: The contralateral foot passes the position of the stance foot.
4. Heel-off: The heel comes off the ground. Higher pressures are present in the metatarsal heads.
5. Toe off: The toes lose their contact with the ground. The least amount of foot is in contact with the surface during this event.
6. Acceleration: quick acceleration of the swinging foot.
7. Midswing: The swinging foot passes the position of the stance foot.
8. Deceleration: The swinging foot decelerates as it approaches the ground. The heel takes its position for a new heel strike.

**Figure 1:**
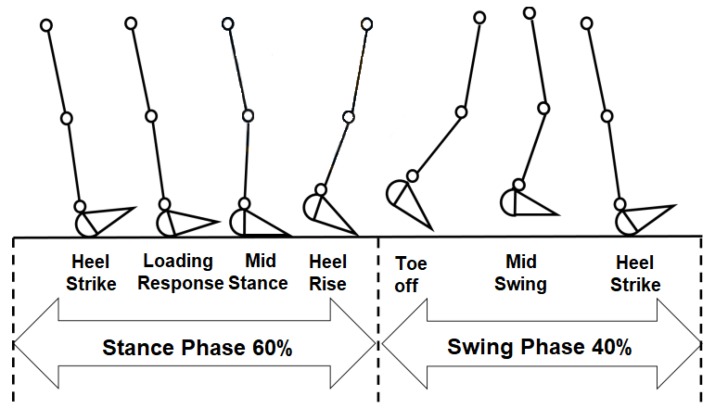
The gait cycle and gait events.

Figure 1 shows a gait cycle and the different events during gait execution. A gait cycle can be divided into two main phases, the stance and swing phase. The stance phase covers approximately 60% of the total cycle, and consists of the instance where the foot keeps contact with the ground. Associated with the previously described events, the first five are executed within the stance phase. On the other hand, the remaining 40% of the cycle belongs to the swing phase. During this phase, the foot is not in contact with the ground; this phase includes events six, seven, and eight [13,14,16].

Analyzing the stance phase, four subphases can be defined, delimited by the events from one to five. The initial contact phase (ICP) occurs when the heel contacts the surface the first time, then the forefoot contact phase (FFCP) starts when the metatarsal heads contact the ground for the first time. Next, the flat foot phase (FFP) is when the middle foot contacts the surface. Lastly, the forefoot push-off phase (FFPOP) starts when the metatarsal heads and the toes are the only support in contact with the ground. The toe-off event is considered the transition between the stance and swing phases [13,17].

The cyclical patterns of gait allow us to obtain parameters and indexes that characterize the gait, which has relevant impacts in clinical applications. If it is possible to define normal ranges of those parameters, these can be characterized as constituting a normal gait. Significant deviations with respect to normal values could indicate the presence of pathological conditions. Using this information, clinicians could have more accurate methods to determine and characterize pathologies and treatment follow-up. Gait is represented by temporal and spatial characteristics. It is possible to analyze these components in order to describe, represent, and/or evaluate the way in which people walk. Temporal components are associated with the time intervals that a person needs to execute each gait phase or subphase. The spatial components refer to relative positions between different segments and/or joints of the lower limbs [18]. There are several ways to measure gait characteristics, using both spatial and temporal indicators. Among the most used methods are:

1. Plantar pressure measurement: This method measures the reaction forces that are generated between the sole of the foot and the ground. To measure this interaction force, transducers are positioned between the support surface and the sole. Several transducers are used for this application: force resistive sensors (FSR), piezoresistive sensors, capacitive sensors, and optoelectronic sensors. Using this method, it is possible to detect gait events by using the sensors as switches and indicating the presence of certain events according to a pressure or non-pressure state. It is also possible to generate pressure distribution maps by quantifying the pressure exerted on the force sensors [3,18].
2. Angular displacement and accelerometry: This method measures and quantifies angular displacement, velocity, and acceleration in different points in the body, usually the lower limbs and trunk. In this application, gyroscopes and/or accelerometers are widely used. Attaching one or more of these sensors to body segments, it is possible to model and describe the movements of lower limbs [18,19].
3. Camera tracking: The methods with cameras use opto-electronic systems to capture the execution of gait in video images. To track body segments, passive reflective markers or active markers such as LEDs are used. With the markers positioned on the body, the system is able to calibrate positions and keep track of movement. Image processing software uses this information to generate a representation of the movement. Its accuracy depends on the number of cameras used and their resolution, which determines the spatial quality of the obtained images [18,20].

Camera-based systems are not suitable to analyze gait in non-laboratory environments. Recent studies compare the usability and acceptance of textile wearables versus angular displacement devices for activity monitoring. As a result, these studies conclude that textile wearables (e.g., smart insoles/socks, smart shirts) are more acceptable and show a better potential for usability in clinical fields than inertial measurement units (IMUs) [21,22]. For these reasons, this paper focuses on a hardware design and gait segmentation algorithm using a wearable pressure-sensing device suitable for daily activity monitoring. In Table 1, different plantar pressure measurement systems and their characteristics are presented.

The most relevant parameters that can be calculated using the pressure-sensing method are listed below.

### 2.1. Vertical Ground-Reaction Forces

This refers to reaction forces produced due to the contact between the plantar surface and the ground. This effect is explained by Newton’s third law. The plantar surface produces a vertical force in the direction of the ground, in response, another force with the same intensity but in the opposite direction is generated [29].

### 2.2. Weight Distribution

This determines the magnitude of the vertical ground-reaction forces as a body weight representation and its distribution in different points of the plantar surface. Weight distribution changes along the gait. It depends on the contact between the plantar surface and the ground in each gait subphase. While standing, toes provide stability, changing the weight distribution. This parameter has relevance in studies of pathologies (diabetes, ACV, Parkinson’s), where is important to identify abnormally high pressures [30,31].

### 2.3. Center of Pressure

**Figure 2:**
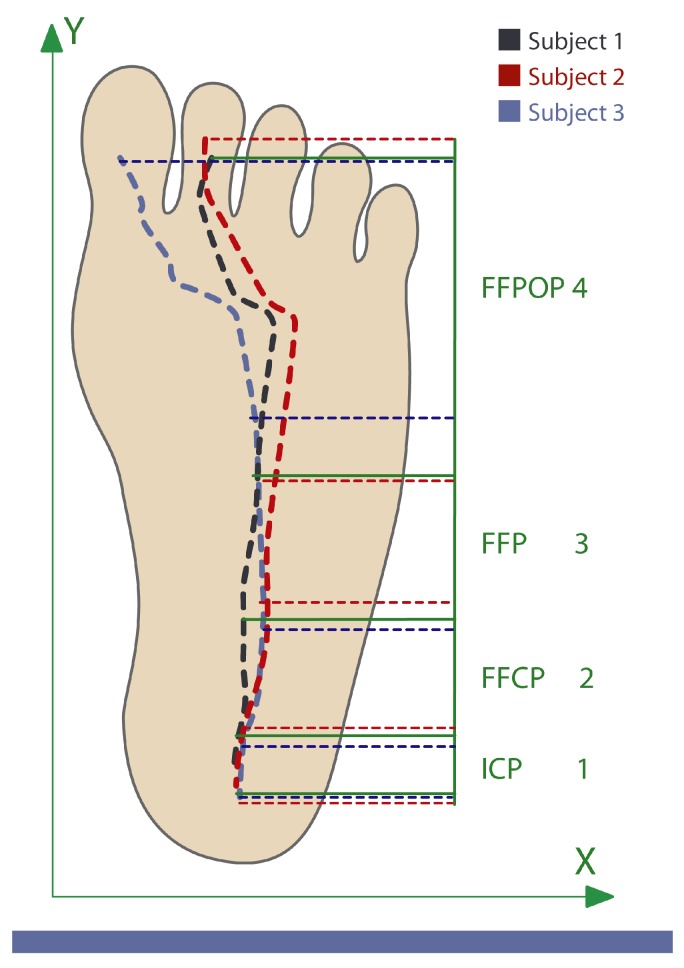
Correlation between center of pressure (CoP) position and gait subphases in the stance phase. Different colors show different subjects’ CoP trajectories.

This is defined as the location in the plane where a resultant force vector is generated. This force represents different forces that are applied in different points of the plane. It is calculated as the mean of all forces weighted by the applied position. It reveals useful information about stability and balance during the gait [13]. The center of pressure (CoP) changes its position along the gait, following patterns associated with gait cycles. Its trajectory begins under the heel then goes straight along the lateral area of the midfoot to the metatarsal zone and ends in the hallux [13]. Different studies have used the CoP to study human gait patterns as it provides biomechanical information associated with gait dynamics. These works propose that CoP position and velocity allows for the segmenting of gait phases and subphases [17,32,33]. Figure 2 shows the relation between CoP trajectory and each subphase of the stance phase [32].

### 2.4. Heel-Strike/Toe-Off Detection

Heel strike and toe off are the two most important events in order to identify the transition between the stance and swing phases. The heel strike determines the beginning of the stance phase and the end of the swing phase. Otherwise, toe off determines the end of the stance phase and the beginning of the swing phase [13,17]. In addition, the change between swing and stance phases determines the beginning of a step. By detecting these events, it is possible to implement simple gait segmentation and step recognition.

### 2.5. Gait Cycle Detection and Segmentation

Gait detection consists in determining and recognizing the different periodic patterns during a gait. Segmentation is the use of those spatial and temporal patterns in order to identify the gait phases and subphases [13,32,34,35]. This allows us to study and characterize the human gait, enabling the identification of normal and pathological conditions [34,35].

### 2.6. Cadence

Corresponds to the steps executed in a segment of time, usually one minute. The normal values of cadence depend on different factors such as age, height, leg length, and gender [13].

## 3. Monitoring System Design

**Figure 3:**
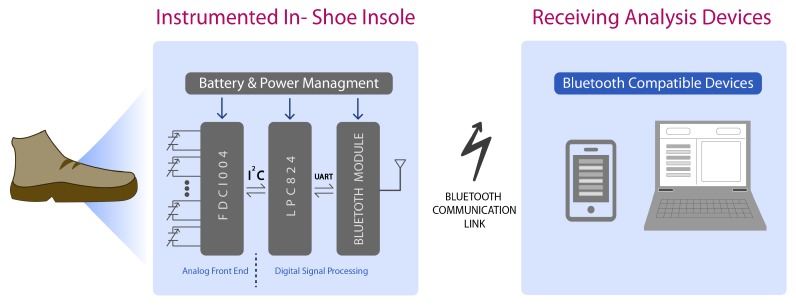
Scheme of system operation.

The proposed system consists of three functional parts: capacitive pressure sensors, acquisition and data transmission hardware, and an external device where algorithms analyze the data. Figure 3 shows a general functional diagram.

### 3.1. Capacitive Pressure Sensors

**Figure 4:**
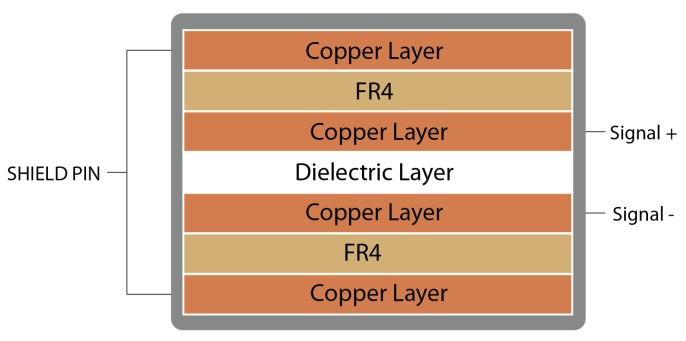
Layer scheme of the designed sensors © 2018 IEEE [36].

Capacitive sensors were manufactured with two superimposed flexible copper films (commercially available flexible PCB from “Fab in a Box”), separated by a flexible dielectric film. The chosen dielectric material was “Electroactive Ferroelectret Film” by EMFIT. This dielectric material was chosen for its properties shown in Table 2, which compared with PDMS, has more adequate properties, making it appropriate for the custom-shape in-shoe capacitive sensor. Its thickness and dielectric permittivity are the main properties that make it suitable and allow for capacitive pressure sensor construction within the required capacity-to-size ratio. This material is pressure compressible, allowing the capacitance to change while returning to its nominal thickness when the pressure is released. The developed capacitive sensors have four copper layers and one dielectric layer (see Figure 4) [36]. The top and bottom copper layers are connected to the shield pin of the capacitance-to-digital converter protecting the sensor from electromagnetic noise and disabling proximity measurement caused by parasitic capacitance through air. The sensor dimensions are 10 mm × 10 mm with a thickness of 0.41 mm. This small sensor size guarantees a single applied pressure over the sensor area as it is smaller than any anatomical zone in the foot.

**Figure 5:**
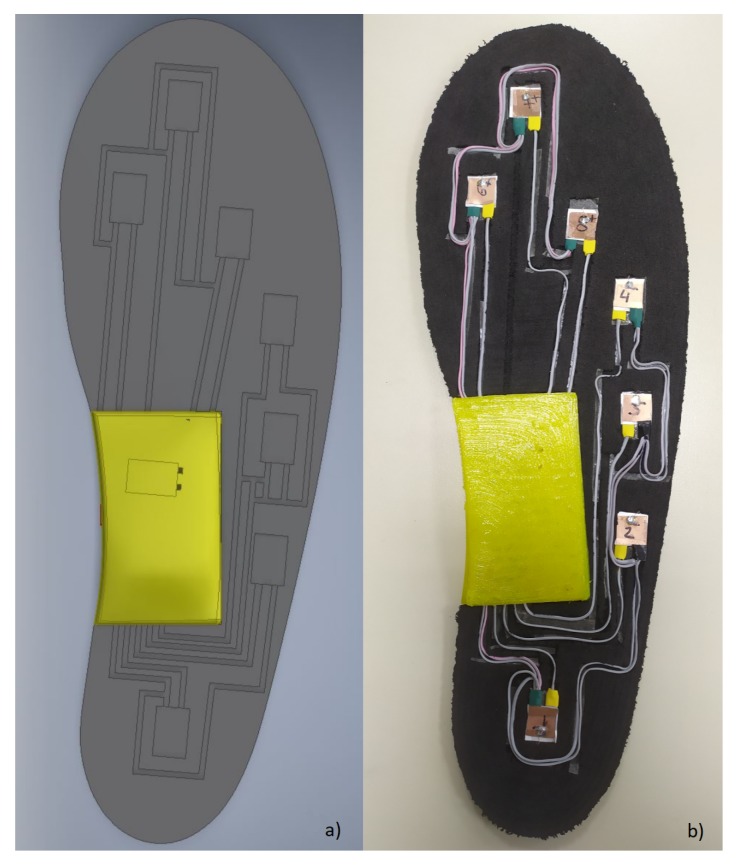
(**a**) 3D design of the insole in AutoDesk Inventor Professional 2018. (**b**) Inside view of the insole with custom-made capacitive sensors located at the main support plantar points.

Sixteen sensors (eight in each insole) were manufactured using an LPKF ProtoLaser S laser structuring machine and then placed in an EVA foam shoe insole. The positions of the sensors were chosen to match the main support and balance plantar points of the body [32]. Figure 5 shows the position of each sensor, located according to the most important support points in the foot [41], in (a) a 3D design of the insole with the positions of the eight sensors and (b) an internal view of the implemented shoe insole with the embedded sensors. The insole was structured by a 5 axis CNC machine (5-axis Makers) and has slots to place sensors in alignment with the plantar surface. In addition, the thin sensor layers totaling a 0.41 mm sensor height are tightly bonded together, minimizing the area changes by displacement.

**Figure 6:**
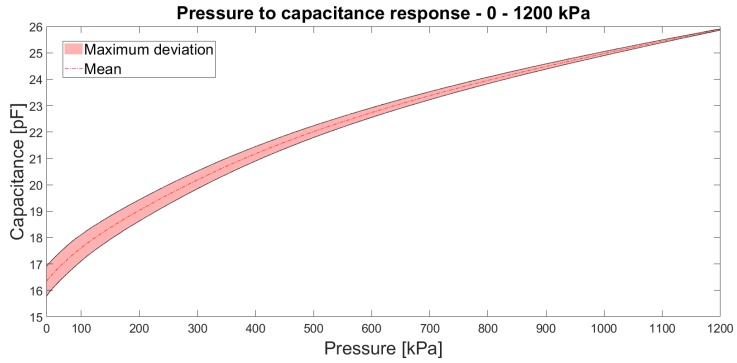
Capacitance-to-force curve of the sensor in the range from 0 to 1200 kPa. The red dashed line shows the mean capacitance value over pressure. The light-red area shows the maximum deviation of capacitance over the pressure range.

The sensors were calibrated using a mechanical press (ProLine table-top Z005 Zwick/Roell). The sensors were submitted to a linear compression force over the total sensor area in the range from 0 to 1200 kPa, three times each. This range of characterization was chosen according to the typical ranges of plantar pressures during walking in the literature [13]. Figure 6 shows the response of the sensors in the tested range. The red dot–dash line represents the mean value for the 3 trials. The red area shows the maximum and minimum values registered during tests. In the tested range, the sensor takes a logarithmic response where the sensor capacitance achieves a total variation of 11 pF (analyzed and tested with Hioki IM-3536 LCR Meter (Hioki E.E. Coporation, Ueda, Nagano, Japan)). However, it can be stated that the sensor responded quite linearly in the range of 500 to 1200 kPa. It is remarkable that the sensor performs well over the whole range of interest. In addition, it can be said that the sensor has good sensitivity for pressures within this range. The temperature response of the sensors was tested in the range from ambient temperature (20${}^{{}^{\circ}}$) to 60${}^{{}^{\circ}}$. The maximum change in capacitance was of −0.8 pF at 60${}^{{}^{\circ}}$ over the whole tested pressure range.

### 3.2. Acquisition Hardware

Two Texas Instruments FDC1004 integrated circuits (ICs) were used to measure sensor capacitance. These ICs communicate by I2C protocol to an NXP micro-controller (MCU). This MCU (LPC824) configures the ICs to adjust the measurement capacitive offset and configures the sample frequency (100 Hz for each channel). During operation, it acquires the pressure measurements with 24 bits of resolution and send them by UART protocol to an ST Microelectronics SPBT3.0DP2 Bluetooth module configured as a serial port profile (SPP) device. The PCB design was done in Autodesk EAGLE 9.4.2 and was manufactured in a 0.8 mm thickness rigid PCB with the LPKF ProtoLaser S laser structuring machine. A Shenzhen Sunbang Technology Co. lithium-polymer (Li-Po) battery of 400 mAh is used to power the system, and a battery charge circuit is included in the PCB design. Digital acquisition hardware PCB was also embedded inside of the EVA foam insole, in the same way as the capacitive sensors. To avoid battery-related risk, the Li-Po battery was carefully placed under the arch area in the foot as the pressure in this anatomic zone is minimal and almost negligible. The sensors are connected to the hardware through electrical wires carefully routed through the EVA foam, avoiding intersection between channels and keeping spacing to reduce parasitic capacitance. In addition, signal+ and signal- wires are wrapped by a shield copper tape electrically connected to the active shield signal. Two instrumented insoles were implemented in order to perform experimental walking pressure validation tests and implement the gait analysis algorithms.

### 3.3. Gait Analysis Parameter Calculation

Calculations to obtain different parameters for gait analysis were implemented, such as CoP coordinates. The algorithms take the plantar pressure signals of each sensor implemented (eight in each insole) as inputs and based on the anatomical locations of the sensors, CoP coordinates are calculated over time. The CoP calculation methodology and gait-phase segmentation algorithm is detailed in the following steps:

#### 3.3.1. Initial Contact and Toe Off Detection

These events mark the beginning and end of the stance phase, respectively. This detection allows us to segment the human gait into stance and swing phases. To achieve this, adaptive thresholds for the heel and the hallux pressure signals are calculated using the moving average between the local maximum and minimum of each signal. Initial contact will be detected when the heel pressure signal exceeds its threshold. On the other hand, the toe-off event is detected when the hallux pressure signal goes below its threshold.

#### 3.3.2. *CoP* Trajectory Calculation

The position of the *CoP* over the total surface was calculated in order to determine the trajectory of the *CoP*. For this, Equations (1) and (2) are used:

(1) $$CoP_{x}=\frac{\sum_{i=1}^{n}X_{i}\cdot P_{i}}{\sum_{i=1}^{n}P_{i}},$$

(2) $$CoP_{y}=\frac{\sum_{i=1}^{n}Y_{i}\cdot P_{i}}{\sum_{i=1}^{n}P_{i}},$$

where *n* is the total number of sensors by insole, $X_{i}$ and $Y_{i}$ correspond to the positions of the sensors in the medial–lateral and anteroposterior axes, respectively (as shown in Figure 5), and finally, $P_{i}$ is the pressure measurement recorded by the *i*th sensor.

#### 3.3.3. Stance Phase Segmentation

To segment the stance phase into the four subphases described in Section 2, the anteroposterior instantaneous *CoP* positions were used. The segmentation was performed by dividing the anteroposterior plane of the foot into four zones that correlate with each subphase. The divisions were considered according to the normal 41 EU foot size (US 10) and the described characteristics of each subphase [32].

#### 3.3.4. *CoP* velocity Calculation

To perform the *CoP* velocity calculation, Equations (3) and (4) are used:

(3) $$VelCoP_{x}=\frac{CoP{\left(T\right)}_{x}-CoP{\left(T-1\right)}_{x}}{0.01},$$

(4) $$VelCoP_{y}=\frac{CoP{\left(T\right)}_{y}-CoP{\left(T-1\right)}_{y}}{0.01},$$

where $CoP{\left(T\right)}_{X}$ correspond to a sample of the *CoP* in the medial–lateral axis and $CoP{\left(T-1\right)}_{X}$ is the previous sample of the *CoP* in the same axis; the same is calculated for the anteroposterior axis. This position difference is divided by 0.01 due to the system sample frequency (100 Hz), obtaining the instant velocity.

#### 3.3.5. Double Support Time

To calculate the period time of the double support phase, the intersection between the times of the stance phase of both feet is realized. The algorithm calculates this intersection using the stance phase time of each foot.

#### 3.3.6. Cadence

Cadence correlates with gait velocity, but its measurement unit is steps per minute. To calculate it, the difference between the detection of initial contact in one foot with the detection in the other foot is used. Then, this calculation is normalized to express it in steps per minute. To calculate cadence, Equation (5) is used:

(5) $$Cadence\;\left(\mathrm{steps}/\min\right)=\frac{Total\;Number\;of\;steps}{Test\;Time\;\left(s\right)}\cdot 60.$$

#### 3.3.7. Gait Phases Percentages

Using all of the previously described indexes, some percentages of the gait-phase times with respect to the total cycle time are calculated. These are the double support time with respect to the total cycle, the stance phase time with respect to the total cycle, and the swing phase time with respect to the total cycle.

## 4. Experimental Results

### 4.1. Energy System Evaluation

**Figure 7:**
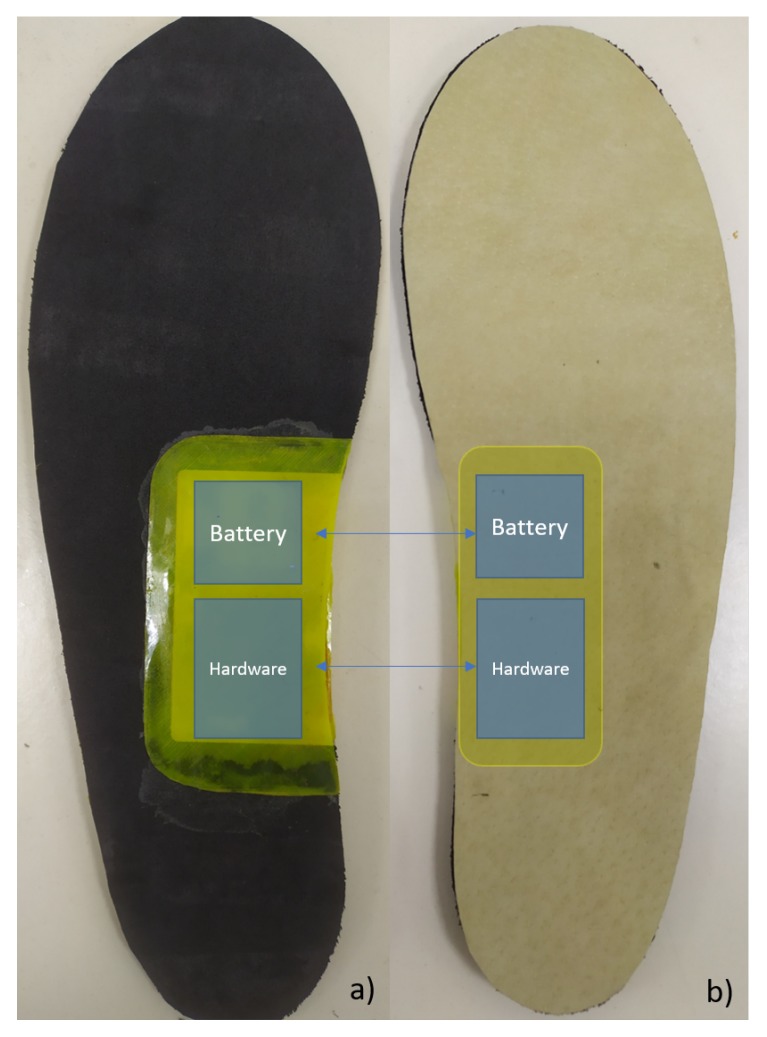
Final developed instrumented insole prototype.

Figure 7 shows the final developed instrumented insole prototype. This system has an average energy consumption of 36 mA on IDLE state and 44 mA while streaming data. The system is energized with a Li-Po battery of 3.7 V, 400 mAh, which gives the device an autonomy of 9 h of continuous operation.

### 4.2. Test Protocol

The system was tested with six healthy male volunteers (age: 24 ± 5 years, weight: 74 ± 7 kg, height: 1.65 ± 0.23 m, EU 41/US 10 foot size) in 10 m walking distance trials. The test consisted in standing still at the beginning of a straight 10 m track then walking across the track at a “normal” (defined by each volunteer) speed and finally standing still at the end for a few seconds. With this simple walking test procedure, the system is capable of analyzing the gait in the most natural way. During the test, participants were asked to wear the instrumented shoe insoles in both shoes. The authorization was requested through informed consent, which was approved by the Biosecurity, Bioethical, and Ethical Committee of the University of Concepción (Number 3180551).

### 4.3. Test Results

**Figure 8:**
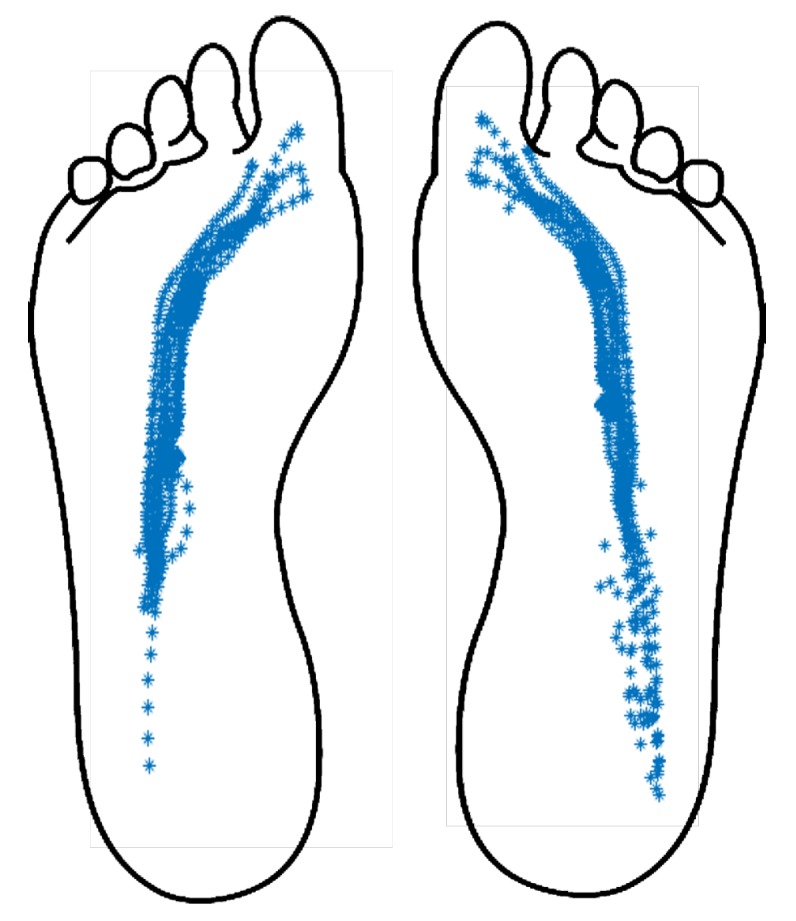
CoP calculated from all gait cycles with the developed system, for one volunteer.

**Figure 9:**
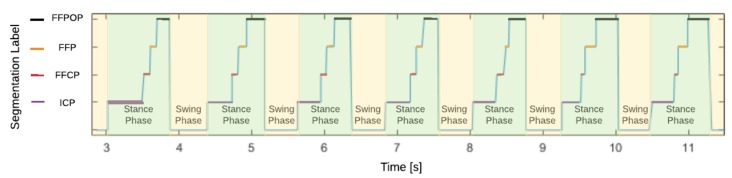
Example of the achieved segmentation of phases and subphases for one volunteer.

**Figure 10:**
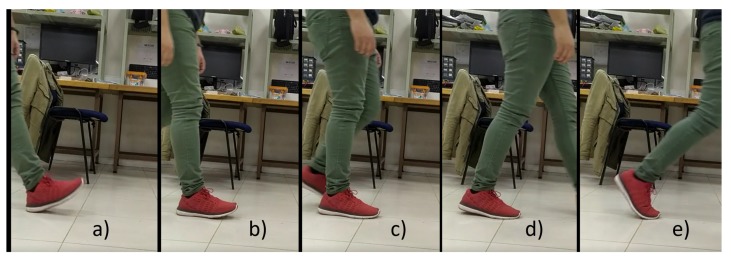
Moments of transition of detected subphases with the implemented algorithm for the foot with the red device. (**a**) Transition from swing phase to ICP. (**b**) Transition from ICP to FFCP. (**c**) Transition from FFCP to FFP. (**d**) Transition from FFP to FFPOP. (**e**) Transition from FFPOP to swing phase.

Using the instrumented shoe insoles with the embedded capacitive sensors, plantar pressure data was registered during the walking test trials. After the trials, no volunteer reported discomfort from wearing the instrumented shoe insoles. With the logged data, the indices described in Section 3.3 were calculated for each volunteer. In Figure 8, an example of CoP trajectory is shown for one of the test trials. In addition, in Figure 9 an extract of 7 steps is shown with the achieved phase segmentation for the same volunteer. The trials were video recorded and analyzed by experts in gait analysis in order to validate the gait segmentation by the proposed algorithm. Figure 10 shows the transitions between each gait phase and subphase from the video recording related to the automatic segmentation of the proposed algorithm. Finally, Table 3 summarizes the obtained results for all indices from all volunteers.

## 5. Discussion

The results show that by using the developed system, it is possible to determine gait events and parameters, as well perform an automated segmentation of the gait cycle phases and subphases. This allows us to evaluate gait symmetry, characterize the gait of a subject, and obtain relevant gait parameters. The main limitation of this preliminary study is the amount of people tested. In order to validate and characterize abnormal gait patterns, an extensive study with more subjects would be needed.

The proposed system (as well as other smart textile devices) has the advantage of enabling a simple gait assessment method with minimal intervention on the user in comparison with multiple camera or IMU systems (camera markers and IMUs in each body segment). In addition, wearable systems enable us to measure gait parameters in a non-confined environment, as required for platforms and camera-based gait laboratories. Even though less slippage and bending issues have been reported in smart socks than in-shoe insoles, they show more variable and noisy signals during static and dynamic performance because of the construction process [42]. On the other hand, this type of system presents the disadvantage of focusing only on plantar pressures, disregarding the contribution of the upper body on gait, thus making it difficult to determine the causes of abnormal gait. Even though these systems do not provide upper body information, they can account for the characterization of gait parameters and assess the evolution of these parameters over time. The achieved analysis may be useful in rehabilitation, allowing objective assessment during the recovery process for patients with gait disorders.

Similar works only detect some events in the gait cycle, such as the toe off, heel lift, and heel strike [8]. Others only identify temporal parameters such as stride time and stance time [9]. The proposed system achieves a successful segmentation of gait phases and subphases, calculates COP trajectory, is capable of reporting COP velocity, double-support time, cadence, stance-phase time percentage, swing-phase time percentage, and double-support time percentage.

Moreover, the detection and classification of the different phases and subphases of gait may be used to compared these with known parameters of healthy subjects in order to assist in pathological condition diagnosis. The implemented system has the major advantage of achieving gait analysis under arbitrary non-controlled environments.

## 6. Conclusions

Capacitive custom-made sensors were designed and manufactured using flexible PCB and EMFIT dielectric sheets. The resulting sensors are 0.41 mm thin with an area of 1 cm^2^. Tests were performed using two instrumented insoles. The volunteers walked 10 m at “normal” gait velocity. Pressure data was recorded and analyzed with the proposed algorithms. The system segmented the gait phases and subphases in all subjects. The calculated percentile distribution between stance-phase time and swing-phase time is almost 60%/40%, which is aligned with literature reports on healthy subjects [13]. In addition, the system correctly calculated the CoP trajectory for healthy subjects, according to literature reports.

## Figures and Tables

**Table 1:** Plantar pressure measurement systems.

	Developer	Format	Type of Sensor	Number of Sensors	Measurement Range (kPa)	Sample Frequency (Hz)
“Automatic identification of gait events using an instrumented sock” [8]	Preece et al.	Sock	Resistive	1	Not registered	1500
“Wearable textile sensor sock for gait analysis” [9]	Tirosh et al.	Sock	Resistive	3	0–240	250
“Development of smart sock system for gate analysis and foot pressure control” [11]	Oks et al.	Sock	Resistive	5	0–500	Not registered
TACTILUS [23]	Sensor Products Inc.	Platform	Piezoresistive	16,384	0.68–1378	1000
FOOTSCAN [24]	RScan International	Platform	Resistive	4096–12,288	10–1270	200
PEDAR [25]	Novel TM	Insole	Capacitive	85–99	>0–1200	50–350
F-SCAN [26]	Novel TM	Insole	Resistive	85–99	345–862	100–750
“Wireless foot plantar pressure measurement instrument for medical diagnostic” [27]	Zizoua et al.	Insole	Resistive	954	Not registered	100
“Development of an in-shoe pressure-sensitive device for gait analysis” [28]	De Rossi et al.	Insole	Optoelectronic	64	>0–1000	100

**Table 2:** Dielectric Material properties.

Property	EMFIT	PDMS
Thickness (μm)	70 [37]	171 to 308 [38]
Operating temp (${}^{{}^{\circ}}$C)	−20 to +50 [37]	−49.9 to +40 [39]
Sensitivity (pC/N)	25 to 250 [40]	Not registered
Density (kg/m${}^{3}$)	330 [40]	0.97 [39]
Dielectric permittivity (at 10 kHz)	1.12 to 1.23 [40]	2.3 to 2.8 [39]

**Table 3:** Gait segmentation and gait index calculation results.

Volunteer	COP Mean Velocity (cm/s)	Mean Double-Support Time (s)	Mean Cadence (step/min)	% Stance Time/Gait Cycle	% Swing Time/Gait Cycle	% Double Support/Gait Cycle
1	2.4406 ± 0.2902	0.3666 ± 0.1405	64.048 ± 0.09929	56.5205 ± 1.4285	43.4795 ± 1.4285	30.9080 ± 10.649
2	2.6487 ± 0.9562	0.3279 ± 0.1549	61.0565 ± 2.9696	57.8156 ± 1.2635	42.1844 ± 1.2635	25.9568 ± 4.8472
3	2.7790 ± 0.1049	0.3961 ± 0.1474	67.2580 ± 2.4875	55.6898 ± 1.5725	44.3102 ± 1.5725	32.4975 ± 1.2264
4	2.5512 ± 0.9462	0.4541 ± 0.1491	73.8115 ± 0.5123	55.4862 ± 3.9318	44.5138 ± 3.9318	42.0178 ± 1.1756
5	3.1203 ± 1.0070	0.3139 ± 0.1346	62.6595 ± 4.1938	60.4598 ± 4.9897	39.5402 ± 4.9897	23.9127 ± 0.9348
6	2.2900 ± 0.9902	0.4776 ± 0.1478	67.0279 ± 3.2851	61.8216 ± 5.9746	38.1784 ± 5.9746	36.8026 ± 1.2419
